# The dual crisis: Examining older adult opioid use and nursing competency

**DOI:** 10.1093/geroni/igaf122.4212

**Published:** 2025-12-31

**Authors:** Noell Rowan, Tamatha Arms

**Affiliations:** University of North Carolina Wilmington, Wilmington, North Carolina, United States; UNCW, Wilmington, North Carolina, United States

## Abstract

The opioid crisis represents a significant public health emergency in the United States, profoundly affecting individuals, families, and healthcare systems alike. Ortiz and colleagues (2022) in a comprehensive meta-analysis revealed that the average percentage of total pain knowledge among nurses was only 52.9% (95% CI: 47.2–58.6). Moreover, Kramarow & Tejada-Vera (2022) found a 320% increase in opioid overdose deaths among older adults and a 500% increase in older adult opioid related hospitalizations. Kaiser (2020) found that 75% of nurse participants felt unprepared to handle an opioid related case. These results underscore the urgent need for improved nurse education and training. The intersection of these challenges—inadequate pain management training, an aging population with complex needs, and the ongoing opioid crisis—creates a perfect storm that demands immediate attention and innovative solutions. This cross-sectional study involved nurse participants recruited through emails that were sent to all LPNs, RNs, and advanced practice nurses in one southeastern state of the United States. Participants completed a Knowledge and Attitudes Survey Regarding Pain (KARSP) tool and a second knowledge survey on opioids. A total of 264 nurses responded, with the majority being nurse practitioners. The mean score was 75% (SD ± 11). Only 45% of the participants understood respiratory depression associated with opioids and 36% were able to identify physical withdrawal symptoms. Implications include improvements and innovative strategies for practitioner education and advocacy for older adults and health care needs.

